# Extrahepatic Comorbidities Associated with Metabolic Dysfunction-Associated Steatotic Liver Disease; A Tertiary Hospital Experience

**DOI:** 10.34172/mejdd.2025.413

**Published:** 2025-04-30

**Authors:** Masoudreza Sohrabi, Mozhdeh Mosalli, Parvin Hassanzadeh, Somayeh Bahrami, Mahmoodreza Khoonsari, Hossein Ajdarkosh, Farhad Zamani

**Affiliations:** ^1^Gastrointestinal and Liver Diseases Research Center, Iran University of Medical Sciences, Tehran, Iran

**Keywords:** Fatty liver disease, Fibrosis, Steatosis, Extrahepatic comorbidities, MASLD

## Abstract

**Background:**

Fatty liver disease and metabolic dysfunction-associated steatotic liver disease (MASLD) have recently been recognized as a major global health issue, and there is much evidence linking MASLD with a range of extrahepatic comorbidities. The current study evaluated extrahepatic comorbidities in patients with MASLD and highlighted the necessity of a multidisciplinary approach to its management.

**Methods:**

A cross-sectional study was conducted at a tertiary center in Tehran. Fatty liver was diagnosed via ultrasonography findings. Exclusion criteria included those with other liver diseases, such as chronic viral hepatitis. Participants underwent liver stiffness measurement using Fibroscan, and clinical data and liver function tests were collected, and questionnaires for DASS-21 and the Pittsburg Sleep Quality Index (PSQI) were completed. We conducted a chi-square test and ANOVA test to evaluate and explore the extrahepatic comorbidities and manifestations associated with fatty liver disease.

**Results:**

Of the 385 study participants, 50.6% had severe steatosis, and 29.1% had severe fibrosis. Several comorbidities, such as hypertension (fibrosis: *P*=0.033, steatosis: *P*=0.011), diabetes (fibrosis: *P*=0.006, steatosis: *P*=0.002), and poor sleep quality (fibrosis: *P*=0.015), were significantly associated with the severity of non-alcoholic fatty liver disease (NAFLD). There were statistically significant differences in mean scores of depression and anxiety across different levels of fibrosis and steatosis (*P*<0.05).

**Conclusion:**

MASLD is a multisystem disorder with widespread extrahepatic manifestations impacting many organs and systems. Patients with MASLD should be screened for comorbidities such as diabetes, cardiovascular disease, and mental health conditions to improve overall health outcomes and prevent disease advancement.

## Introduction

 Metabolic dysfunction-associated steatotic liver disease (MASLD), formerly known as non-alcoholic fatty liver disease (NAFLD), is one of the most common chronic liver diseases worldwide. It occurs in the absence of other contributing factors, such as alcohol consumption, medications, or other chronic liver conditions that can lead to liver fat accumulation, affecting more than one-third of the global population.

 Importantly, MASLD is a complex disorder influenced by various cardiometabolic and environmental risk factors.^1-4^ It encompasses a spectrum of liver dysfunction, ranging from benign steatosis to metabolic dysfunction-associated steatohepatitis, which can progress to liver fibrosis, cirrhosis, and its complications.^5,6^ The prevalence of MASLD in the general population almost same as NAFLD and is estimated at approximately 38%, with even higher rates among individuals who are obese or diabetic. MASLD has now become the leading indication for liver transplantation in the United States.^7,8^

 MASLD is often considered the hepatic manifestation of metabolic syndrome (MetS), and the main cause of death in these patients remains cardiovascular diseases.^9,10^ MASLD is also associated with an increased risk of extrahepatic complications, including chronic kidney disease, type 2 diabetes, sarcopenia, and various extrahepatic cancers. Beyond its physical health impacts, MASLD also affects social and daily life. It is linked to fatigue, reduced health-related quality of life, decreased work productivity, greater healthcare utilization, and a significant economic problem.^1,5,11-15^

 For the past decade, substantial evidence has connected MASLD to a variety of extrahepatic conditions, including type 2 diabetes, chronic kidney disease, atherosclerosis, and cardiovascular disease.^9,16^ The causal relationship between MASLD and these conditions remains unclear. It is uncertain whether MASLD directly contributes to or exacerbates their development. Furthermore, fatty liver is associated with a growing list of conditions, such as cardiomyopathy, cardiac arrhythmias, osteoporosis, obstructive sleep apnea, polycystic ovarian syndrome, male sexual dysfunction, psoriasis, hypothyroidism, urolithiasis, and periodontitis.^1,17,18^ As a result, healthcare providers and patients need to recognize the multi-organ involvement of fatty liver and MASLD, which can manifest without a clear clinical pattern or sequence. A high level of clinical suspicion based on a patient’s risk profile is essential, as widespread screening for MASLD is not currently recommended. This study aimed to explore the extrahepatic comorbidities and manifestations associated with fatty liver disease. This work did not look for a cause-effect relationship.

## Material and Methods

 A cross-sectional study was conducted between March 2022 and December 2023, with participants selected from patients referred to the liver clinic at the Gastrointestinal and Liver Disease Research Center, Firoozgar Hospital, Tehran, Iran. The required sample size was estimated to be 385 patients using the Cochran formula. The study included patients aged 18 years or older who had no history of liver surgery, bariatric surgery, or use of medications that affect the liver, such as silymarin, Ursobil, or statins. Patients with other known causes of liver disease, including chronic hepatitis B and C, hemochromatosis, autoimmune hepatitis, and Wilson’s disease, were excluded. NAFLD was diagnosed based on the presence of fatty liver observed in ultrasonographic evaluations, with no other underlying hepatopathies.

###  Measurements

####  Fibroscan

 All participating patients underwent liver fibroscan (Fibroscan 502, Paris, France) in our center. An expert physician performed the Fibroscan. Hepatic fat was quantified using the controlled attenuation parameter (CAP) in decibels per meter (dB/m), while fibrosis was assessed via liver stiffness measurement in kilopascals (kPa). Regarding the results, fibrosis is categorized as mild, moderate, and severe, which are < 6.5 kPa, 6,5-10 kPa, and > 10 kPa, respectively. Indeed, regarding steatosis, CAP less than 240, 240-300, and more than 301 are considered mild, moderate, and severe steatosis, respectively.^19^

####  Sleep Quality Assessment

 Sleep quality was evaluated based on the Persian version of the Pittsburgh Sleep Quality Index (PSQI-P).^20,21^ The PSQI is a commonly used tool to assess sleep quality in the past month and consists of 19 questions that measure seven components of subjective sleep quality, sleep latency, sleep duration, typical sleep efficiency, sleep disturbance, use of sleep medications, and daytime dysfunction.^22^ Previous studies have demonstrated the reliability and validity of the PSQI-P. In the study by Khorami Rad and colleagues, the reliability of the questionnaire was reported with Cronbach’s alpha of 0.82, and the intraclass correlation coefficient (ICC) was 0.87.^22,23^ Each component was assigned a score from 0 (no problem) to 3 (very serious problem), and finally, the scores of each component were added together, resulting in a global PSQI score ranging from 0 to 21. A high score in each component or in the total score indicates poor sleep quality. A total PSQI score greater than 5 indicates poor sleep quality.^24^

####  DASS-21 Assessment

 To assess mood, the Persian version of the validated Depression, Anxiety, and Stress Scale (DASS-21) questionnaire was used.^25^ This questionnaire includes three components of stress, depression, and anxiety, each of which is measured by seven questions. Each question is assigned a score from 0 to 3. The score of each component includes the sum of the scores of the questions that make up that component. The total score for each component ranges from 0 to 21. Since this questionnaire is a shortened form of its 42 questions, the final score of each subscale was multiplied by 2.^26,27^

###  Clinical and Demographic Data Collection

 Each patient underwent an interview and clinical examination conducted by two expert gastroenterologists. A comprehensive questionnaire was completed for each patient, including demographic and anthropometric data, physical examination findings, and laboratory results.

 Weight was measured using a scale to the nearest 100 grams, with the patient wearing light clothing and no shoes. Height was measured to the nearest 0.5 cm using a stadiometer. Body mass index (BMI) was calculated by dividing weight (in kg) by the square of height (in meters). Waist circumference (WC) was measured at the midpoint between the uppermost border of the iliac crest and the lower border of the costal margin (rib cage). Patients were also asked about their sleep quality and quantity concerning the PSQI.

 The factors for assessing sleep quality included falling asleep in less than 15 minutes, staying asleep without the use of sleep medications, absence of sleep disturbances such as sleepwalking or apnea, lack of light or fragmented sleep, and sleeping between 10:00 PM and 4:00 AM. Sleep quantity was measured in terms of hours of sleep per night. The DASS-21 included 21 self-reporting questions.

 Liver function tests, including alkaline phosphatase (ALP), serum albumin, aspartate aminotransferase (AST), bilirubin, alanine aminotransferase (ALT), platelet count, international normalized ratio (INR), total cholesterol (Chol), triglycerides (TGs), high-density lipoprotein (HDL) cholesterol, low-density lipoprotein (LDL) cholesterol, and hemoglobin A1c (HbA1c), were performed by the central laboratory of Firoozgar Hospital. Diabetes mellitus^28^ and hypertension were diagnosed according to the criteria set by the American Diabetes Association (ADA) and the Joint National Committee (JNC), respectively. Blood pressure was measured in three positions (lying, sitting, and standing).

###  Statistical Analysis

 Data analysis was done using SPSS software for the Windows (IBM-SPSS, version 26.0, Chicago, IL, USA). Descriptive statistics were used to summarize the data, with continuous variables reported as means ± standard deviation (SD) and categorical variables presented as frequencies and percentages. The association between qualitative variables and outcomes was examined using the chi-square test, and the relationship between quantitative variables and outcomes was examined using the ANOVA test.

## Results

 A total of 385 patients with mild to severe fatty liver disease were included in this study. Of them, 201 (52.2%) were male. The mean age of the participants was 45.83 ± 12.25 years. Additionally, the mean of BMI was 29.67 ± 5.15. The basic characteristics of the patients are illustrated in Table 1.

**Table 1:** Demographic characteristics of patients with MASLD in the study

**Variable**	**Subgroup**	**Male** ** No. (%)**	**Female ** **No. (%)**	**Total ** **No. (%)**
Age (y)	18-25	10 (5.0)	3 (1.6)	13 (3.4)
	26-45	96 (47.8)	68 (37.0)	164 (42.5)
	46-65	85 (42.3)	102 (55.4)	187 (48.6)
	> 65	10 (5.0)	11 (6.0)	21 (5.5)
Marital status	Single	42 (20.9)	36 (19.6)	78 (20.3)
	Married	159 (79.1)	148 (80.4)	307 (79.7)
Smoking	Yes	61 (30.3)	42 (22.8)	103 (26.8)
	No	140 (69.7)	142 (77.2)	296 (73.2)
Physical activity^a^	None	90 (56.6)	77 (52.7)	167 (54.8)
	Yes	69 (43.4)	69 (47.3)	138 (45.2)
BMI (kg/m^2^)	18.5-24.9	38 (18.9)	40 (21.7)	78 (20.34)
	25-29.9	86 (42.8)	49 (26.6)	135 (35.1)
	30-34.9	53 (26.4)	59 (32.1)	112 (29.1)
	35-39.9	24 (11.9)	36 (19.6)	60 (15.6)
Sleep Quality^b^	Good	99 (49.3)	87 (47.3)	186 (48.3)
	Poor	100 (49.8)	96 (52.2)	196 (50.9)
Depression^c^	Normal	121 (60.2)	100 (54.3)	221 (57.4)
	Mild	54 (26.9)	61 (33.2)	115 (29.9)
	Moderate	14 (7.0)	11 (6.0)	25 (6.5)
	Severe	12 (6.0)	12 (6.5)	24 (6.2)
Anxiety^d^	Normal	54 (26.9)	54 (29.3)	108 (28.1)
	Mild	26 (12.9)	33 (17.9)	59 (15.3)
	Moderate	58 (28.9)	52 (28.3)	110 (28.6)
	Severe	63 (31.3)	45 (24.5)	108 (28.1)
Stress^e^	Normal	52 (25.9)	55 (29.9)	107 (27.8)
	Mild	40 (19.9)	39 (21.2)	79 (20.5)
	Moderate	72 (35.8)	54 (29.3)	126 (32.7)
	Severe	37 (18.4)	36 (19.6)	73 (19.0)
Diabetes^f^	Yes	21 (16.9)	31 (26.7)	52 (21.7)
	No	103 (83.1)	85 (73.3)	188 (78.3)
High blood pressure	Yes	20 (16.0)	29 (24.6)	49 (20.2)
	No	105 (84.0)	89 (75.4)	194 (79.8)
Gastrointestinal disease^g^	Yes	24 (19.2)	14 (11.9)	38 (15.6)
	No	101 (80.8)	104 (88.1)	205 (84.4)
Cancer	Yes	13 (10.4)	24 (20.3)	37 (15.2)
	No	112 (89.6)	94 (79.7)	206 (84.8)
Fibrosis^h^	Mild	45 (22.4)	47 (25.5)	92 (23.9)
	Moderate	91 (45.3)	90 (48.9)	181 (47.0)
	Severe	65 (32.3)	47 (25.5)	112 (29.1)
Steatosis^i^	Mild	23 (11.4)	29 (15.8)	52 (13.5)
	Moderate	73 (36.3)	65 (35.3)	138 (35.8)
	Severe	105 (52.2)	90 (48.9)	195 (50.6)
		**Mean±SD**	**Mean±SD**	**Mean±SD**
Abdominal obesity (cm)		102.91 ± 14.45	102.27 ± 15.6	102.61 ± 15.0
Actual Sleep Hours		5.91 ± 1.73	6.01 ± 1.78	6.01 ± 1.79

BMI: body mass index. **a.** Walking at least half an hour a day, **b. **Sleep Quality: Good < 5, Poor > 5, **c. **Depression: normal (0-9), mild (10-13), moderate (14-20), severe and very severe < 21, **d.** Anxiety: normal (0-7), mild (8-9), moderate (10-14), severe and very severe ( < 15), **e.** Stress: normal (0-14), mild (15-18), moderate (19-25), severe and very severe ( < 26), Missing data: **f**; n = 145,** g;** n = 142. **h.** Mild < 6.5, Moderate (6.5-10), Severe > 10. **i.** Mild < 240 dB/m, Moderate (240 –300) dB/m,Severe > 301dB/m.

 The average severity of fibrosis in patients was 8.20 ± 3.65. In female patients, it was 7.90 ± 3.30, and in male patients, it was 8.50 ± 3.30. The average rate of steatosis in patients was 294.41 ± 51.68. In female patients, it was 289.45 ± 52.07, and in male patients, it was 298.95 ± 51.02. As illustrated in Tables 2 and 3, liver enzymes, total cholesterol, and LDL cholesterol were associated with increasing fibrosis and steatosis.

**Table 2:** Examining lab factor scores in fibrosis levels of patients with MASLD

**Variable**	**Subgroup**^a^	**Mean±SD**	**Mean±SD (Total)**	***P*** ** value**	**Normal range **
FBS (mg/dL)	Mild	109.85 ± 15.85	117.43 ± 72.20	0.307	60-125
	Moderate	113 ± 37.22			
	Severe	133.08 ± 59.92			
ALT (U/L)	Mild	38.23 ± 57.24	55.32 ± 47.54	< 0.001*	7-55
	Moderate	59.71 ± 42.82			
	Severe	66.12 ± 38.27			
AST (U/L)	Mild	30.42 ± 27.96	45.85 ± 32.08	< 0.001*	5-40
	Moderate	49.86 ± 33.06			
	Severe	55.85 ± 28.85			
TG (mg/dL)	Mild	123.22 ± 66.59	164.84 ± 117.81	< 0.001*	< 150
	Moderate	197.71 ± 145.26			
	Severe	160 ± 90.41			
CHOL (mg/dL)	Mild	168.43 ± 44.78	184.82 ± 52.70	< 0.001*	< 200
	Moderate	203.22 ± 52.66			
	Severe	169.59 ± 53.58			
HDL (mg/dL)	Mild	43.83 ± 15.68	44.37 ± 27.70	0.886	35-65
	Moderate	44 ± 14.70			
	Severe	46.24 ± 55.61			
LDL (mg/dL)	Mild	109.84 ± 32.66	117.39 ± 35.41	< 0.004*	< 100
	Moderate	126.04 ± 38.39			
	Severe	111.34 ± 28.75			

**a: **Mild < 6.5 kPa, Moderate (6.5 - 10) kPa, Severe > 10 kPa. * Significant at the < 0.05 level.

**Table 3:** Examining lab factor scores in steatosis levels of patients with MASLD

**Variable**	**Subgroup**^a^	**Mean±SD**	**Mean±SD (Total)**	***P*** ** value**	**Normal range **
FBS (mg/dL)	Mild	90.59 ± 9.18	117.43 ± 72.20	0.092	60-125
	Moderate	127.04 ± 110.89			
	Severe	120.51 ± 47.82			
ALT (U/L)	Mild	34.16 ± 32.55	55.32 ± 47.54	< 0.001*	7-55
	Moderate	51.47 ± 43.94			
	Severe	65.53 ± 41.19			
AST (U/L)	Mild	28.50 ± 22.83	45.85 ± 32.08	< 0.001*	5-40
	Moderate	43.53 ± 34.09			
	Severe	53.58 ± 30.72			
TG (mg/dL)	Mild	112.97 ± 69.72	164.84 ± 117.81	0.002*	< 150
	Moderate	155.85 ± 95.73			
	Severe	189.81 ± 138.98			
CHOL (mg/dL)	Mild	153.05 ± 48.93	184.82 ± 52.90	< 0.001*	< 200
	Moderate	184.41 ± 54.58			
	Severe	195.84 ± 48.74			
HDL (mg/dL)	Mild	43.45 ± 8.80	44.37 ± 27.70	0.639	35-65
	Moderate	42.45 ± 8.38			
	Severe	46.21 ± 39.46			
LDL (mg/dL)	Mild	102.05 ± 30.47	117.39 ± 35.41	0.019*	< 100
	Moderate	121.04 ± 40.12			
	Severe	1.65 ± 31.60			

**a.** Mild < 240 dB/m, Moderate (240 –300) dB/m,Severe > 301dB/m * Significant at the < 0.05 level.

 Regarding Tables 4 and 5, having diabetes, age, BMI, high WC, depression, and anxiety are associated with developing fibrosis and increasing steatosis.

**Table 4:** Examining the relationship between patient characteristics and fibrosis levels

**Variable**	**Fibrosis**^a^			***P*** ** value**
	**Mild** **n (%)**	**Moderate** **n (%)**	**Severe** **n (%)**	
Sex, Male	45 (48.9)	91 (50.3)	65 (58)	0.334
Physical Activity, yes	48 (69.6)	62 (41.9)	28 (31.8)	< 0.001*
Smoking, yes	27 (29.3)	47 (26)	29 (25.9)	0.812
Diabetes, yes	7 (12.7)	19 (17.6)	26 (33.8)	0.006*
Hypertension, yes	5 (8.9)	23 (20.9)	21 (27.3)	0.033*
Kidney failure, yes	2 (3.6)	4 (3.6)	4 (2.5)	0.845
Gastrointestinal disease, yes	9 (16.1)	17 (15.5)	12 (15.6)	1
Cancer, yes	5 (8.9)	20 (18.2)	12 (15.6)	0.280
Sleep quality, poor	58 (63)	89 (49.2)	49 (45)	0.015*
	**Mean±SD**	**Mean±SD**	**Mean±SD**	
Age (y)	38.58 ± 11.12	47.96 ± 11.03	48.35 ± 12.78	< 0.001*
BMI (kg/m^2^)	25.56 ± 4.00	30.51 ± 4.66	31.68 ± 4.92	< 0.001*
Abdominal obesity (cm)	93.38 ± 13.54	103.72 ± 13.12	108.38 ± 15.55	< 0.001*
Actual sleep hours	5.34 ± 1.86	6.05 ± 1.76	6.30 ± 1.53	< 0.001*
Depression	15.81 ± 18.42	11.55 ± 12.48	11.58 ± 11.58	0.039*
Anxiety	52.52 ± 9.48	20.85 ± 9.19	22.67 ± 9.46	0.039*
Stress	20.29 ± 7.84	18.46 ± 7.39	18.88 ± 7.60	0.166

BMI: body mass index. **a: **Mild < 6.5 kPa, Moderate (6.5-10) kPa, Severe > 10 kPa. Bolded values are significant at the < 0.05. * Significant at the < 0.05 level.

**Table 5:** Examining the relationship between patient characteristics and steatosis levels

**Variable**	**Steatosis**^a^			***P*** ** value**
	**Mild** **n (%)**	**Moderate** **n (%)**	**Severe** **n (%)**	
Sex, Male	23 (44.2)	73 (52.9)	105 (53.8)	0.458
Physical activity, yes	21 (77.8)	47 (56.6)	70 (35.9)	< 0.001*
Smoking, yes	11 (21.2)	44 (31.9)	48 (24.6)	0.208
Diabetes, yes	2 (4.9)	15 (19.0)	35 (29.2)	0.002*
Hypertension, yes	5 (11.9)	10 (12.7)	34 (27.9)	0.011*
Gastrointestinal disease, yes	7 (16.7)	11 (13.9)	20 (16.4)	0.877
Cancer, yes	6 (14.3)	6 (7.6)	25 (20.5)	0.045*
Sleep quality, poor	29 (55.8)	71 (52.2)	96 (49.5)	0.691
	**Mean±SD**	**Mean±SD**	**Mean±SD**	
Age (y)	37.48 ± 10.24	44.18 ± 11.67	49.23 ± 11.88	< 0.001*
BMI (kg/m^2^)	24.32 ± 4.58	29.29 ± 5.08	31.37 ± 4.25	< 0.001*
Abdominal obesity (cm)	90.46 ± 12.37	101.54 ± 14.96	106.60 ± 13.80	< 0.001*
Actual sleep hours	5.93 ± 1.67	5.55 ± 1.78	6.25 ± 1.72	0.002*
Depression	11.84 ± 12.96	15.39 ± 16.31	10.78 ± 12.08	0.011*
Anxiety	28.15 ± 7.35	22.26 ± 10.07	21.15 ± 9.25	< 0.001*
Stress	21.46 ± 7.37	19.04 ± 7.17	18.36 ± 7.81	0.032*

BMI: body mass index. **a.** Mild < 240 dB/m, Moderate (240 –300) dB/m,Severe > 301dB/m * Significant at the < 0.05 level.

 With respect to DASS 21, we found mild, moderate, and severe depression were present in 30%, 6.5%, and 2.6% of participants. 29.3% and 33.9% of patients with moderate and severe fibrosis complained of mild depression. Indeed, anxiety significantly increased by advancing fibrosis. There was a statistically significant relationship between the levels of sleep quality and levels of fibrosis (Table 4), so 45% of patients with severe fibrosis, 49.2% of patients with moderate fibrosis, and 63% of patients with mild fibrosis reported poor sleep quality. Moreover, there was no statistically significant relationship between sleep quality and steatosis levels (Table 5).

## Discussion

 Identifying extrahepatic comorbidities in MASLD is essential for a comprehensive approach to disease management, as its impact extends beyond liver-related complications. Given the systemic nature of MASLD, addressing its multifaceted effects requires a multidisciplinary strategy.

 This study examines the extrahepatic comorbidities associated with MASLD, without establishing a cause-and-effect relationship through a cross-sectional analysis of patients. Our findings highlight key associations between MASLD, comorbidities, and lifestyle factors, which can influence both disease progression and management. It is important to emphasize that MASLD can develop even without noticeable changes in liver enzyme levels. Moreover, elevated liver enzymes do not necessarily indicate the severity of the underlying disease.^29,30^ Hence, identifying patients with simple steatosis and MASLD to prevent major morbidity and eventual mortality has become a critical issue. In this context, it has been demonstrated that MASLD may be associated with comorbidities even before fibrosis develops. Furthermore, MASLD could potentially act as a precursor for the development of MetS components in the future.^31^ Therefore, MASLD may play a crucial role in disrupting body homeostasis, highlighting the need for greater attention to its prevention and early detection.

 Regarding our findings, age emerged as a significant factor influencing disease severity, with older patients more likely to exhibit advanced stages of fibrosis and steatosis. In this context, the onset of fatty liver disease is a critical factor, as it can progress to severe liver damage and its associated complications.^32-34^ An epidemiologic study revealed that age more than 50 years was associated with more disabilities and high fasting glucose levels related to MASLD.^30^ Moreover, another study demonstrated that age was related to the progress of MASLD, and the peak of its prevalence could be observed in patients aged more than 45 years.^34^

 Regarding BMI, the study revealed a high prevalence of overweight or obesity, a well-documented risk factor for MASLD. Consistent with previous research, a higher BMI was associated with increased severity of both liver fibrosis and steatosis, underscoring the significant role of obesity in the development of MASLD.^35,36^ Son and colleagues in a study on middle-aged patients, found that BMI was a statistically significant predictor of MASLD, with every 1 kg/m^2^ increase in BMI raising the likelihood of MASLD by 1.14-fold.^10^ Additionally, the analysis showed that male patients had slightly higher levels of fibrosis and steatosis compared with females, a finding that aligns with other studies highlighting sex differences in MASLD progression.^37^ Furthermore, it has been demonstrated that globally, the age-standardized disability-adjusted life-years rate for high fasting plasma glucose-attributable MASLD is consistently higher in men than in women.^30^

 This difference may be linked to sexual hormones, as females are often protected from dysmetabolism due to hormonal factors. Additionally, animal models have shown that estrogen deficiency can contribute to hepatic inflammation, potentially facilitating disease progression.^38-40^

 This underscores the cumulative nature of liver damage linked to MASLD and highlights the critical importance of early detection and management to prevent long-term complications.^33,41^ According to the World Obesity Federation’s 2023 report, it is estimated that 1 in 4 people (approximately 2 billion individuals) will have obesity by 2035. During the same period, childhood obesity is projected to double. Notably, obesity is rising at an accelerated rate in low-income countries, further emphasizing the urgency of addressing this global health challenge.^42^ Additionally, abdominal obesity was significantly associated with both fibrosis and steatosis, reinforcing the central role of visceral fat in the pathogenesis of MASLD and its associated comorbidities.^41^

 Furthermore, our study reinforced the strong relationship between MASLD and MetS, as several markers of MetS, such as hypertension, diabetes, and dyslipidemia, were closely associated with the progression of liver disease. MASLD can be considered a component of MetS due to this strong association. It is widely accepted that MASLD is linked to insulin resistance, a condition that independently increases cardiovascular risk.^43,44^

 In particular, the study revealed a higher prevalence of diabetes and hypertension in patients with severe fibrosis, suggesting that these conditions may exacerbate liver damage. The association between elevated TG and LDL levels with more severe fibrosis and steatosis further highlights the cardiovascular risks associated with MASLD. These findings align with the well-established hypothesis that MASLD is not merely a liver disease but also a significant risk factor for cardiovascular diseases, including atherosclerosis and myocardial infarction.^43-46^

 The research revealed an intriguing association linking depression and anxiety to higher levels of fibrosis and steatosis, suggesting a potential bidirectional relationship. Although no significant direct link was found between depression and fibrosis, individuals with moderate to severe fibrosis exhibited elevated depressive symptoms. These findings are consistent with prior studies indicating that the emotional burden of chronic conditions like MASLD may exacerbate disease progression, particularly when combined with poor sleep quality, a common issue among these patients.^28,47^

 One of the critical issues, particularly among young adults, is sleep quality. Recent studies have highlighted changes in both sleep duration and quality.^48,49^ In this context, sleep quality showed a significant correlation with the severity of both fibrosis and steatosis, with a notably higher prevalence of poor sleep quality among individuals with severe fibrosis. This finding suggests that disruptions in sleep patterns may contribute to the progression of MASLD. The link between inadequate sleep and liver disease could be mediated by metabolic imbalances, such as insulin resistance and increased sympathetic nervous system activity, which are frequently observed in patients with MASLD.^50,51^ However, the lack of a significant association between sleep quality and steatosis levels underscores the need for further research to better understand the complexities of this relationship. It remains to be determined whether improving sleep quality could help mitigate liver damage in individuals with MASLD.

 In addition to cardiovascular diseases and metabolic abnormalities, MASLD has been associated with various extrahepatic conditions, including gastrointestinal diseases, cancer, and kidney disease.^52-54^ The link between MASLD and cancer, particularly colorectal and hepatocellular carcinoma, has been widely studied. Research suggests that cancer occurrences are more prevalent in individuals with severe steatosis, indicating that MASLD may act as a risk factor for cancer, potentially due to chronic inflammation and oxidative stress associated with liver fat accumulation.^55,56^ Furthermore, the increased prevalence of gastrointestinal disorders and kidney disease among patients with MASLD underscores the need for further research to better understand the complex interactions between these systems.

## Limitations

 While the study is valuable in providing further insights into the comorbid health conditions associated with MASLD, it is not without limitations. The study’s cross-sectional design hinders making causal conclusions, as the observed relationships could be influenced by unaccounted confounding variables. Longitudinal studies will be needed the temporal dynamics of these associations and their consequences for long-term health.

## Conclusion

 MASLD is not just a liver disease; it is a systemic condition with extrahepatic effects that impact multiple organs and systems. The findings of this study highlight the importance of early detection and a multidisciplinary approach to managing both the hepatic and extrahepatic aspects of the disease. To improve overall health outcomes and prevent disease progression, patients with MASLD should be screened for comorbidities such as diabetes, cardiovascular disease, and mental health disorders.
